# Video based heart rate detection in unrestrained laboratory rats: a feasibility analysis

**DOI:** 10.1038/s41598-025-25816-5

**Published:** 2025-10-30

**Authors:** Julianna Monissen, Lucas Mösch, Matthias Monissen, Laura Warner, Ute Lindauer, Michael Czaplik, Carina Barbosa Pereira

**Affiliations:** 1 Department of Anaesthesiology, Faculty of Medicine, RWTH Aachen University, Pauwelsstraße 30, 52074 Aachen, NRW Germany; 2 Department of Neurosurgery, Faculty of Medicine, RWTH Aachen University, Pauwelsstraße 30, 52074 Aachen, NRW Germany

**Keywords:** Laboratory animals, Animal welfare, RPPG, Contactless heart rate, Biological techniques, Engineering, Physiology

## Abstract

Continuous monitoring of vital signs in laboratory animals is often essential for reliable scientific results and severity assessment. It still depends on invasive approaches such as transponder implementation, which affect the animals well-being. To minimize this impact, a camera-based method is proposed for heart rate detection by analyzing skin color variations in unrestrained rats. Pulse signals were extracted from video recordings taken with a smart home cage designed in a preceding study and processed using classical signal processing methods. For the successfully detected heart rates, we achieved a mean absolute error (MAE) of **10.6 beats per minute (bpm)** and a root mean square error (RMSE) of **13.8 bpm**. The results suggest that heart rate detection is feasible. Despite challenges such as difficult lighting conditions and small regions of interest, this study demonstrates the potential for non-invasive heart rate monitoring in freely moving laboratory animals.

## Introduction

While experimentation on animals remains an indispensable component of medical research^1^, ensuring the welfare of laboratory animals through rigorous and humane assessment methods is crucial for both ethical considerations and the validity of scientific outcomes^2–4^. The European Directive 2010/63/EU^5^ is the regulatory framework for all animal research in the European Union. It aims to promote the highest standards of animal welfare while allowing research necessary for advancing knowledge and protecting human and animal health. The directive requires that alternatives to animal testing are used whenever possible and mandates strict regulations for the care and use of animals in experiments. Additionally, it encourages the implementation of the 3Rs principle - Replacement, reduction, and refinement, first introduced by Russell and Burch^6^.

To be able to estimate the pain and distress of laboratory animals objective, classifiable, and standardized severity assessment parameters are necessary^7,8^. Well-being can be evaluated with multiple psychological and physiological parameters such as appearance, body metrics (weight, temperature, heart rate (HR)heart rate (HR), environment (e.g. nest quality), and behavior^9^. HR and HR variability (HRV) have been recognized as valuable parameters for stress and well-being^10–12^. Traditional measurement methods often involve direct skin contact with animals or even sensor implantation^13,14^. These procedures have an impact on animal welfare and, consecutively, may influence and bias the outcome of research studies^15^.

Rodents are extensively utilized in research due to their notable anatomical, physiological, and genetic resemblance to humans, coupled with their manageable size and ease of maintenance^16^. They account for approximately half of all experimental animals used in trials^17^. For accurate welfare examinations, it is essential that the animals remain conscious and unrestrained during monitoring. This approach minimizes stress induced by handling or restraint, thereby providing more reliable data regarding their natural physiological state.

This underscores the need for innovative approaches that minimize disturbance to the animals while providing accurate data. Radar is one of the techniques that was applied to monitor cardiorespiratory movement of rats^18^ and with radio frequency near-field coherent sensing vital signs of small conscious animals in their laboratory living quarters were measured. Breathing and heartbeat curves could be successfully obtained but reference data for validation lacked^19^.

Remote Photoplethysmography (rPPG) is a further contactless technique that has already been applied in humans and animals for monitoring HR. It is a camera based approach, that operates on the premise that subtle changes in skin color, imperceptible to the naked eye, occur as a result of fluctuations in blood volume caused by the heartbeat. These changes in color are captured using an RGB camera^20^.

In humans there are already many studies that could detect HR from the face^21^, using classical computer vision and deep learning. In brief, these studies showed that the HR can be accurately monitored using rPPG under white light illumination and with moving subjects^21^.

Currently, there are not many publications focusing on video-based monitoring of HR in rodents. Wang et al. reported on HR detection of a resting and an anesthetized pig^22^. The Region of Interest (RoI)s were selected manually and conventional computer vision and signal processing methods were applied. In the two recordings HR could be detected with mean absolute error (MAE) of 4.69bpm.

In 2019, Kunczik et al. published an article focusing on assessment of achr in rodents, both mice and rats, using a RGB camera^23^. The video-based HR detection in this setup was possible but the animal were shaved and anesthetized. The HR was detected by tracking multiple pixels on the animal and extracting a movement signal with principle component analysis (PCA).

Previous camera-based approaches have demonstrated rodent heart-rate estimation under controlled conditions, including high-speed imaging of the rat’s sole with bedding removed and bright, uniform illumination, achieving ECG-level agreement^24^, and motion-based methods that track corner motions and suppress respiratory harmonics to derive achr with low relative error in well-lit environments^25^.

Although the subtle changes in skin color occur in rodents, it is not straightforward to translate the algorithms applied in humans to animal research. New challenges arise with laboratory rodents because lighting conditions and physiology are considerably different. There are few hairless body parts where skin can be analyzed and the RoIs are much smaller than in humans or pigs.

This study complements existing camera-based approaches by demonstrating color-based rPPG under welfare-friendly, bedding-equipped home-cage conditions with infrared illumination and continuous operation, using a lower frame rate (40 fps) and multi-site RoIs (ear, tail, paw). This paper presents an rPPG method for the assessment of HR of conscious, unrestrained rats within the home cage. The videos analyzed in this paper were recorded from animal trials in which the smart home cage, published in^26^, was used. Here, we aim at demonstrating the feasibility of the proposed approach as well as highlighting the various factors within the recording setup and signal processing steps that can influence the accuracy and reliability of the results.

## Materials and methods

### Data acquisition

Video data of rats was acquired with a smart home cage setup proposed by Mösch et al.^26^. Two near infrared (NIR) sensitive RGB cameras of the type Pi Camera Module 2 noIR recorded the rodents. The near-infrared sensitivity of the camera led to a pink tone of the videos. The frame rate of the cameras was 40 fps and their resolution was 1632x928pixels. To accurately extract a rat’s HR, which can reach up to 600 bpm (10 Hz), the frame rate must be more than twice this frequency, as per the Nyquist-Shannon sampling theorem. Therefore, a minimum of 20 frames per second (fps) was required. Given that the provided frame rate was 40 fps, it was more than sufficient to ensure accurate extraction and reconstruction of the pulse signal.

**Fig. 1:**
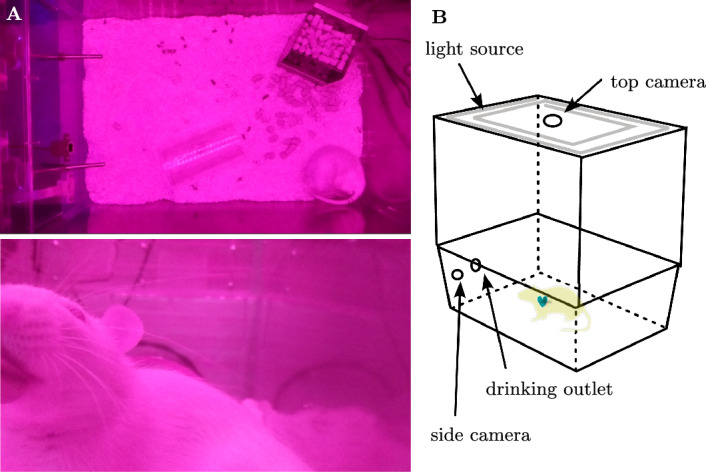
Recordings and recording setup. (**A**) Standstill recordings top view and side view. (**B**) Home Cage setup^26^.

One of the cameras was mounted on top of the cage, visualizing the whole cage area (Fig. 1A top). The second camera was mounted on the side next to the drinking water outlet. It covered only a part of the cage, thus the animals were only in sight temporarily. A standstill example of a frontal recording is shown in Fig. 1a, bottom. The camera positioning is visualized in Fig. 1B.

Since the recordings took place in a low-light environment without ambient light an external 850 nm NIR light source was installed. Since NIR light is able to enter tissue layers beneath the epidermis, it can be used to capture color changes due to varying hemoglobin concentration.

#### Study design

Five male Wistar rats were used in a study to examine subarachnoid hemorrhage (SAH) using the cisterna magna injection model (injection of 300 ${\frac{\upmu l}{min}}$). Approval for this study was obtained from the State Office for Consumer Protection and Nutrition of North Rhine-Westphalia (LAVE, Recklinghausen, Germany, reference number 81-02.04.04.2017.A457), which oversees compliance with the German Animal Welfare Act and the EU Directive 2010/63.

Three of the rats were implanted with an electrocardiogram (ECG) transponder (DSI Model HD-S11-F2). The electrode cables were attached to the pectoralis muscle and to the muscles in the area of the Processus xiphoideus. The ECG was recorded with Ponemah Version 6.32 from DSI and was used in this study as ground truth.

Table 1 summarizes the experimental procedure. The animals were delivered and then placed in the home cages. After an adaption phase to the new environment for approx. 14 days, the transponders were implanted, which measured the ECG among other parameters. After two weeks, the SAH surgery was performed. Animal 1 and animal 2 were completely naive animals (neither transponder implantation nor SAH surgery). Animals 3, 4 and 5 received transponder implantation and subsequently underwent the SAH surgery, performed in the acute setup previously described by Conzen et al.^27^, here adapted for the chronic model. In animal 3 and animal 4, the SAH was triggered with 300$\upmu$ l of blood, which was injected within one minute. Animal 5 received all surgical steps, but no SAH was triggered here (sham control).

The main part of videos analyzed here contained a single rat (animal 3). A small analysis for generalisability was made on recordings of animals 4 and 5. The analyzed recordings were made between 01.05.2023 and 12.05.2023. Recordings were started every 10 minutes and lasted for 30 s. For top view recordings motion detection was used to skip recordings if the animals changed positions. Small movements, such as ear scratching, were not detected. During the recordings, only a single rat was in the home cage. The cage contained a drinking outlet, bedding, nestlests, a food dispenser, and a transparent tube.

**Table 1:** Timeline for experiments.

Animal	Pre transponder		Post transponder		Pre SAH		Post SAH	
	Start	End	Start	End	Start	End	Start	End
1					31.03.2023	01.04.2023	03.04.2023	13.04.2023
2					31.03.2023	01.04.2023	03.04.2023	13.04.2023
3	14.04.2023	16.04.2023	17.04.2023	30.04.2023	30.04.2023	01.05.2023	02.05.2023	12.05.2023
4	28.04.2023	30.04.2023	01.05.2023	12.05.2023	12.05.2023	14.05.2023	15.05.2023	25.05.2023
5	28.04.2023	30.04.2023	01.05.2023	12.05.2023	12.05.2023	14.05.2023	15.05.2023	25.05.2023

#### Generation of skin color intensity signals

**Fig. 2:**
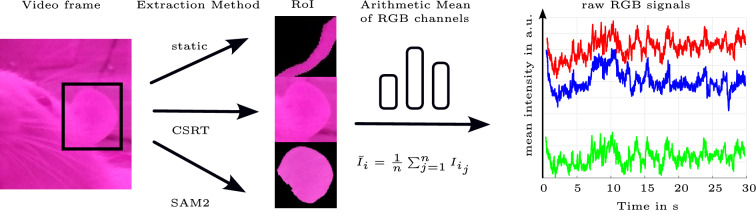
Graphical representation of the approach used to extract mean intensity signals from the acquired video frames. All steps were repeated for each video frame to receive the raw signal in time domain.

The rPPG signal extraction process is visualized in Fig. 2. The HR was assessed based on the skin color changes due to blood flow. Hence, regions on the animal with visible skin were analyzed. Those regions are called RoI and they were selected manually. To retrieve a color signal from the videos, the tails, ears, and paws, visible in the video, were tracked. Two approaches for tracking the body parts were used. The first approach was using a Channel and Spatial Reliability Tracking (CSRT) tracker from the openCV library based on^28^. To remove the background in the resulting RoI, thresholding was applied only if it did not misclassify pixels within the target object as background; otherwise, thresholding was not applied. The second approach was using Facebook’s Segment Anything Model 2 (SAM2)^29^. The model was employed to select the target body part, create a mask and track it throughout the video. For sleeping rats, a static polygonal RoI was manually defined.

For a frame rate of 40 fps, the total number of video frames analyzed per video clip was 1200. The light intensity signals $s_\textrm{R}(t)$, $s_\textrm{G}(t)$, and $s_\textrm{B}(t)$ were gained from the RoI by calculating the mean light intensity values of each color channel, respectively: $\bar{I_i} = \frac{1}{n} \sum _{j=1}^{n} I_{i_j} \, i \in \{R,G,B\}$, where $I_i$ is the light intensity and *n* is the number of pixels per RoI. The averaging allows to reduce the image noise induced by the camera. The same was done for the RoI of the background, which was used for noise reduction in a later step.

#### Heart rate detection

**Fig. 3:**
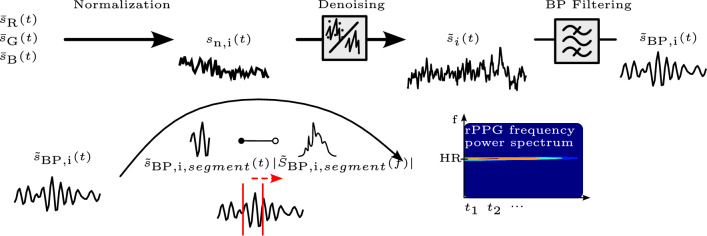
Signal Processing Pipeline.

The signal processing steps are visualized in Fig. 3. The signal of each channel was normalized using equation 1,

1 $$\begin{aligned} s_\textrm{norm,i}(t) = \frac{s_\textrm{i}(t)}{\upmu (s_\textrm{i}(t))}-1, \, i \in \{R,G,B\}, \end{aligned}$$

where $s_\textrm{norm,i}(t)$ is the normalized signal of channel *i* and $\upmu (s_i(t))$ is the temporal mean over a signal length of *n* samples (one sample per frame): $\upmu (s_i(t)) = \frac{1}{n} \sum _{j=1}^{n} s_{i_j}(t) \, i \in \{R,G,B\}$.

Different denoising techniques were applied to the signals $s_i(t)$ and $s_\textrm{norm,i}(t)$. The denoised signal is denoted as $\tilde{s}_i(t)$. A finite impulse response (FIR) bandpass (BP) filter was applied to $\tilde{s}_i(t)$ to remove baseline wander and noise outside the desired frequency band. The cutoff frequencies were 300 bpm and 600 bpm. The resulting signal was denoted as $\tilde{s}_{\textrm{BP}_i}(t)$.

$\tilde{S}_i(f)$ was obtained by dividing $\tilde{s}_{\textrm{BP}_i}(t)$ in segments with a length of 5 s to 20 s with 1 s overlap and by calculating the Fast Fourier Transform (FFT) of each segment. Zero-padding with four times the signal length zeros was utilized to increase the number of frequency bins. The power spectrum was computed for each denoising method and the best result was chosen. The best result was selected based on frequency strength and unambiguity. Finally, the HR was determined by choosing the most powerful frequency in the frequency power spectrum. Each video yielded a scalar HR. A HR was considered successfully detected if it was within ± 30 bpm of the reference HR. Because no published standard exists for rPPG accuracy in laboratory rodents, we specified an a priori, expert-defined tolerance of ± 30 bpm. This value reflects a pragmatic starting point for feasibility assessment and corresponds to approximately 5–10% of the heart-rate levels observed in our recordings (300 to 600 bpm).

Different methods were applied for noise reduction, namely Normalized Least Mean Square (NLMS) adaptive filter^30^, spectral denoising, principle component analysis (PCA), and independent component analysis (ICA). PCA was performed both on the whole signal and on the segmented signal, with segment length of 200 samples (5 s). The noise signal gained from the background RoI $n_{\textrm{norm,i}}(t)$ was used for NLMS filtering and spectral denoising. For spectral denoising the magnitude spectra $|N_{\textrm{norm,i}}(f)| = |\mathcal {F}\{n_\textrm{norm,i}(t)\}|$ and $|S_{\textrm{norm,i}}(f)| = |\mathcal {F}\{s_\textrm{norm,i}(t)\}|$ were computed, respectively. A threshold based on the maximum value in the noise magnitude spectrum was set: $T = \max (|N_{\textrm{norm,i}}(f)|)$. An attenuation factor was defined as

2 $$\begin{aligned} A(f) = {\left\{ \begin{array}{ll} 1 & ; |S_{\textrm{norm,i}}(f)|>= T \\ 0 & ; |S_{\textrm{norm,i}}(f)| < T \end{array}\right. }, \end{aligned}$$

and applied to preserve significant frequencies and suppress noise: $\tilde{S}_{\textrm{norm,i}}(f) = S_{\textrm{norm,i}}(f) \cdot A(f)$. The denoised signal was obtained by performing the inverse Fourier Transform: $\tilde{s}_i(t) = \mathcal {F}^{-1}\left\{ \tilde{S}_{\textrm{norm,i}}(f)\right\}$.

Only the red and blue color channels were used for heart rate analysis, because the green channel did not contain any useful information, due to limited ambient light.

#### Choice of RoI

**Fig. 4:**
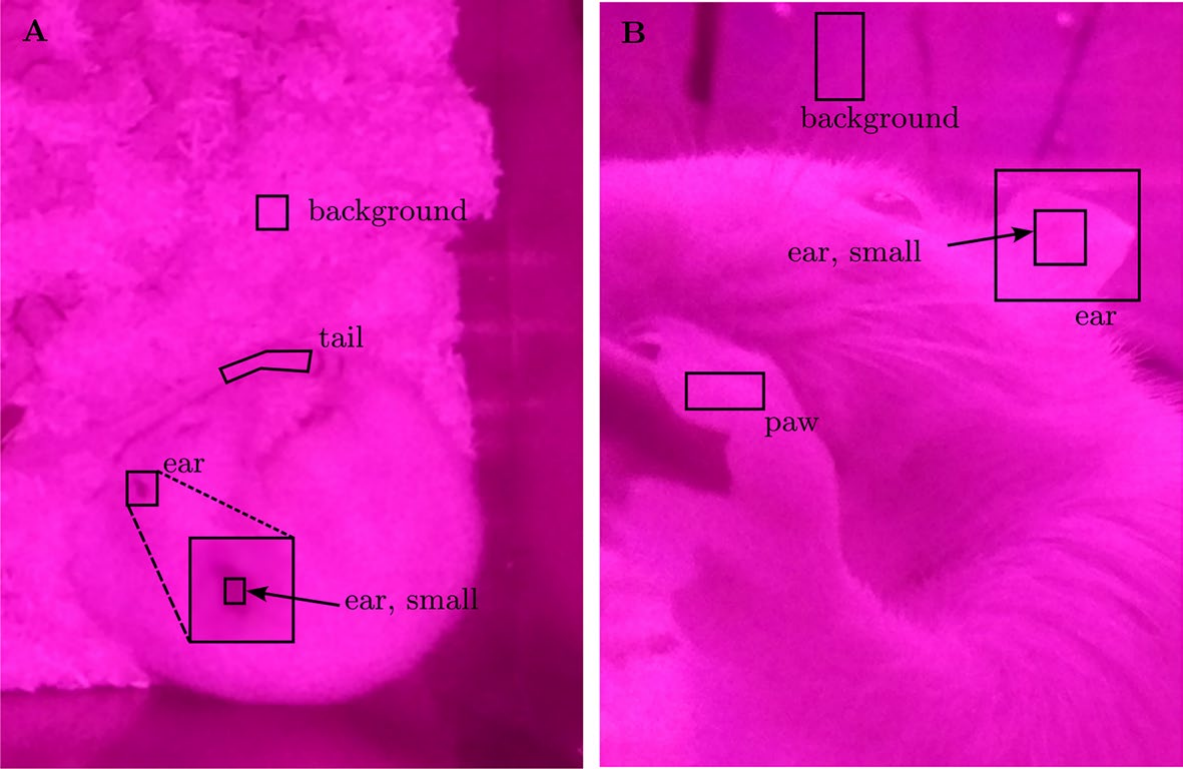
Example of RoI selections. A: Example of RoI selection in top view video. B: Example of RoI selection in side view video.

The rPPG can only be recovered from regions with visible skin. Therefore, the nose, tail, paws, and ears were suitable body regions for achr detection. Examples for the selection of RoIs are shown in Fig. 4. Video segments with minimal movement of the rat were chosen (e.g., during sleeping, drinking, or periods of inactivity). During sleep, only small motion artifacts were expected, movements such as changing the position of the paw, tail, or head occurred. Unpredictable movements of the paw frequently occurred while the rat was drinking. If the rat moved during the recording, the body part of interest was tracked as described above.

For denoising purposes, an additional RoI in the background was selected. Two viable regions for this purpose were the bedding and the cage wall. When opting for the bedding, the RoI had to be positioned sufficiently far from the rat to prevent any vibrations of the rat’s heartbeat from being captured in the background RoI. Otherwise, the pulse signal could be lost during signal denoising. Conversely, when selecting an RoI on the cage wall, it could be placed in close proximity to the laboratory animal, as long as there was no reflection of the animal.

## Results

In this section, the data are reviewed and three different outcomes are described. First, the accuracy of the proposed approach across the dataset is presented. Second, the quality of RoI extraction using the different methods (static, CSRT and SAM2) is compared. Third, an exemplary extracted signal is analyzed in both time and frequency domains, and compared with the ground truth (achr extracted from the ECG). Finally, using a subset of signals, the time resolution achieved with our approach is demonstrated.

### Data review and database description

Only a subset of recordings was suitable for HR detection, since the rat could move freely in the smart home cage. Therefore, there were video sequences where the rat was out of the camera’s reach, e.g. hidden under the bedding or in the transparent tube. In some cases, even though the animal was visible, the specific body parts for achr extraction were not visible, or their movements were too strong, causing massive artifacts on the signal to be extracted.

Table 2 provides an overview of the amount of valuable data captured in the home cage setup. As mentioned previously, only recordings showing the tail, ears, or paws were suitable for analysis. The proportion of video data featuring the rat sleeping was also noteworthy, as the analysis of such recordings was less affected by motion artifacts. It was evident that the top view provided a substantial amount of quality data, while in the side view, the rat was frequently not on the focal point of the camera. Nevertheless, the side-view recordings were valuable, as the rat was usually close to the camera when visible, allowing for a detailed analysis of its skin.

**Table 2:** Visibility of rats in video recordings.

	Top view	Side view
Total number of reviewed videos*	497	2580
Rats were sleeping in	56%	–**
Tail was visible in	79%	0.3%
Ear was visible in	45%	4.3%
Paw was visible in	21%	0.3%
Any furless bodypart was visible in	88%	5%

* Fewer top view videos were analyzed compared to side view due to differing visibility of the rats in these settings, ensuring numbers represent the entire dataset.

** In recordings suitable for analysis, the rat was consistently awake.

Each camera viewpoint had its advantages and disadvantages. In the side view recordings the rat was very close to the camera, enabling the use of larger RoIs with a higher signal-to-noise ratio. In contrast, the rat was awake in all useful recordings, again introducing more motion artifacts. The increased movement of the conscious rat required precise tracking. Overall, in the side view camera recordings the rat was visible in only 5% of the recordings.

In most of the top view recordings at least one hairless body part was visible, making them suitable for HR analysis. In addition, the rat was visible while sleeping, which was favorable since a lot less motion artifacts were introduced into the signal. However, the rat was at a greater distance from the camera, which reduced the signal-to-noise ratio and made it more difficult to find a suitable RoI.

To apply the method to a smaller database, a subset of recordings was chosen based on the best visibility of the ear, tail, or paw. Overall, the database for this work consists of 129 videos, 96 top view recordings were analyzed, and 33 side view recordings. They produced 95 mean intensity signals. A coherent signal could be obtained from 74% of the RoIs. In some cases, the tracker failed due to strong movement of the rat. This led to a number of unsuccessful signal extractions. The number of rPPG signals analyzed was further reduced due to a limited availability of referencee ECG data.

#### Assessing HR detection across different extraction methods and anatomical regions

The overall success rates are summarized in Table 3. It compares the different signal extraction methods, as well as body parts, and color channels. The success rate was calculated by dividing the number of signals in which the HR was successfully determined by the total number of signals within the category. For the color channels, the success rate represents the proportion of signals across all categories where the HR was successfully determined. The HR could be successfully detected in **61.3 % to 81.8 %** of the extracted signals, depending on the signal extraction method. Since the tail was rarely visible in side view recordings and the paws in any recordings (see Table 2) only occasional signals could be evaluated. SAM2 led to a slightly higher success rate than static RoI extraction in the top view recordings for tails. In the top view recordings, tail RoIs had higher success rates than ear RoIs. In the side view recordings higher success rates could be reached than in the top view recordings. CSRT led to higher success rates than SAM2. Except for signals extracted with SAM2 in the top view recordings the success rates are similar in the red and blue color channels. Considering only successfully detected HR (within ± 30 bpm of reference), the Mean Absolute Error (MAE) of static and CSRT extraction was **10 bpm**, and the Root Mean Square Error (RMSE) was **13 bpm**. HR from signals extracted with SAM 2 had a MAE of **11 bpm** and a RMSE of **3.4 bpm**. All methods combined achieved a MAE of **10.6 bpm** and a RMSE of **13.8 bpm**.

**Table 3:** Results of frequency analysis in video data of rats: Number of signals (*N*) and success rate (SR) in % for the categories extraction method, different body parts and color channel. Success rate was determined by dividing the number of successfull achr detections by the number of analysed signals in that category. E.g. $SR = \frac{N_{\textrm{HR detectedTail}}}{N_{\textrm{Tail}}}$, where $N_{\textrm{HR detectedTail}}$ is the number of signals retrieved from tails where the achr was successfully detected, and $N_{\textrm{Tail}}$ the number of signals retrieved from tails.

	Static (top)		SAM (top)		CSRT (side)		SAM (side)	
	*N*	SR	*N*	SR	*N*	SR	*N*	SR
tail	**32**	**65.6**	**20**	**65.0**	0	0	0	0
ear	14	57.1	3	33.3	**10**	**80.0**	**11**	**72.7**
paw	3	33.3	0		1	100	1	0
sum	49	61.3	23	61.9	11	81.8	12	72.7
red	49	36.4	23	64.3	11	61.9	12	63.6
blue	49	33.3	23	38.1	11	55.6	12	63.6

**Fig. 5:**
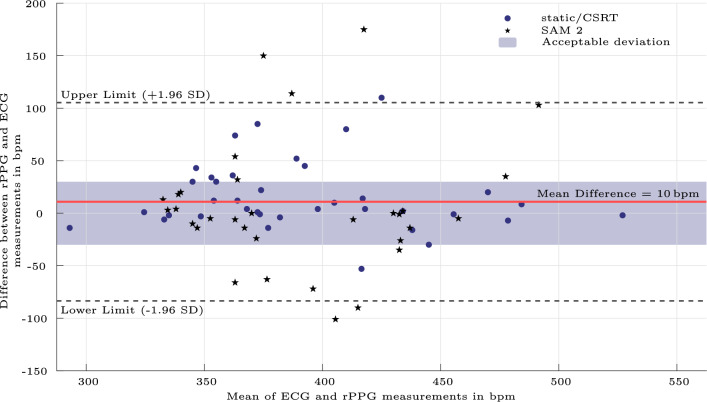
Bland-Altman plot comparing rPPG and ECG measurements. The solid line represents the mean difference (bias), and the dashed lines indicate the limits of agreement (mean ± 1.96 SD). Acceptable deviation of ± 30 bpm is highlighted by a shaded band.

To evaluate the agreement between the reference ECG measurements and the non-invasive rPPG measurements, the Bland-Altman method^31^ was utilized. The Bland-Altman plot in Fig. 5 shows a mean difference (bias) of **10 bpm**, with limits of agreement from**−83 bpm** to **+105 bpm**, when combining both datasets. For the extraction methods static and CSRT the bias is **15 bpm**. For the ECG versus rPPG measurements extracted with SAM 2, the mean difference was **5 bpm**. The majority of data points fell within the ± 30 bpm tolerance threshold, as indicated by the shaded band on the plots. A small number of data points lay outside the limits of agreement. The HR detection was valid over the whole frequency range. Signals in which no HR could be detected are not covered.

#### Characteristic regions of interest and resulting color intensity signals

**Fig. 6:**
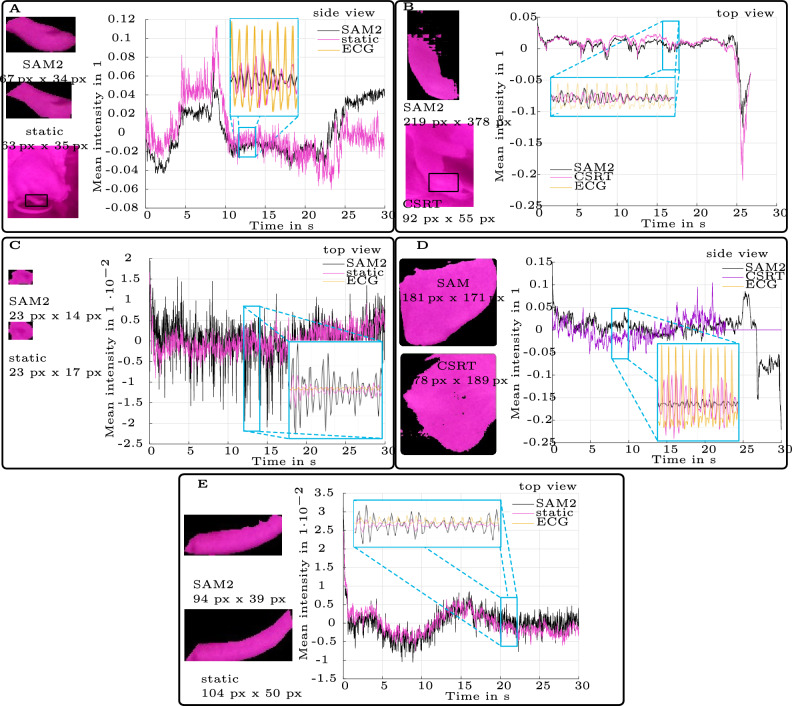
RoIs and resulting mean intensity signals. RoIs gained with extraction methods static (**A,C,E**), CSRT (**B,D**), and SAM2 (all) from top camera recordings (**A,C,E**) and side camera recordings (**B,D**). RoI sizes differ across body parts. The close ups contain the filtered rPPG $\tilde{s}_{\textrm{BP}_i}(t)$ and the ECG is scaled to the rPPG signals. A: Paw: SAM 2 vs. static. The whole paw is segmented with SAM2, only one part with static RoI. Heart rate could be detected in rPPG signal extracted with static RoI, not SAM2. B: Paw: SAM2 vs. CSRT. The whole paw is segmented with SAM2, only one finger with CSRT. Heart rate could be detected in rPPG signal extracted with CSRT, not SAM2. C: Ear: SAM2 vs. static. Clear RoI size differences between body parts and side/top recordings are visible. D: Ear: SAM2 vs. CSRT. Clear RoI size differences between body parts and side/top recordings are visible. E: Tail: SAM 2 vs. static. With SAM2 clear outlines of the tails are not given in most cases.

In Fig. 6 a selection of RoIs with their resulting signals $s_\textrm{B,norm}(t)$ is shown (normalized mean value of the RoI for the blue channel). It demonstrates characteristic features of the RoI and rPPG signals resulting from the different extraction methods static, CSRT, and SAM2. A static RoI was only used in top view recodings, because the rat was still enough. CSRT was only used in side view recordings, because the rat moved in each usable recording.

SAM 2 provided signals with reduced baseline drift compared to both a static ROI and the CSRT tracker. However, when the rat was lying still, a static ROI could produce signals comparable to those of SAM 2 (example 6A). The static ROI offered a clear selection of the tail, which was not achieved with SAM 2 (example 6E). The RoI size was not crucial for good signal quality.

In side view recordings with significant movement of the rat, SAM 2 delivered signals with fewer distortions compared to CSRT (example 6D). Additionally, SAM 2 maintained a better object tracking than CSRT under certain conditions when the focus was lost (example 6D).

#### High-fidelity HR detection in single video analysis

**Fig. 7:**
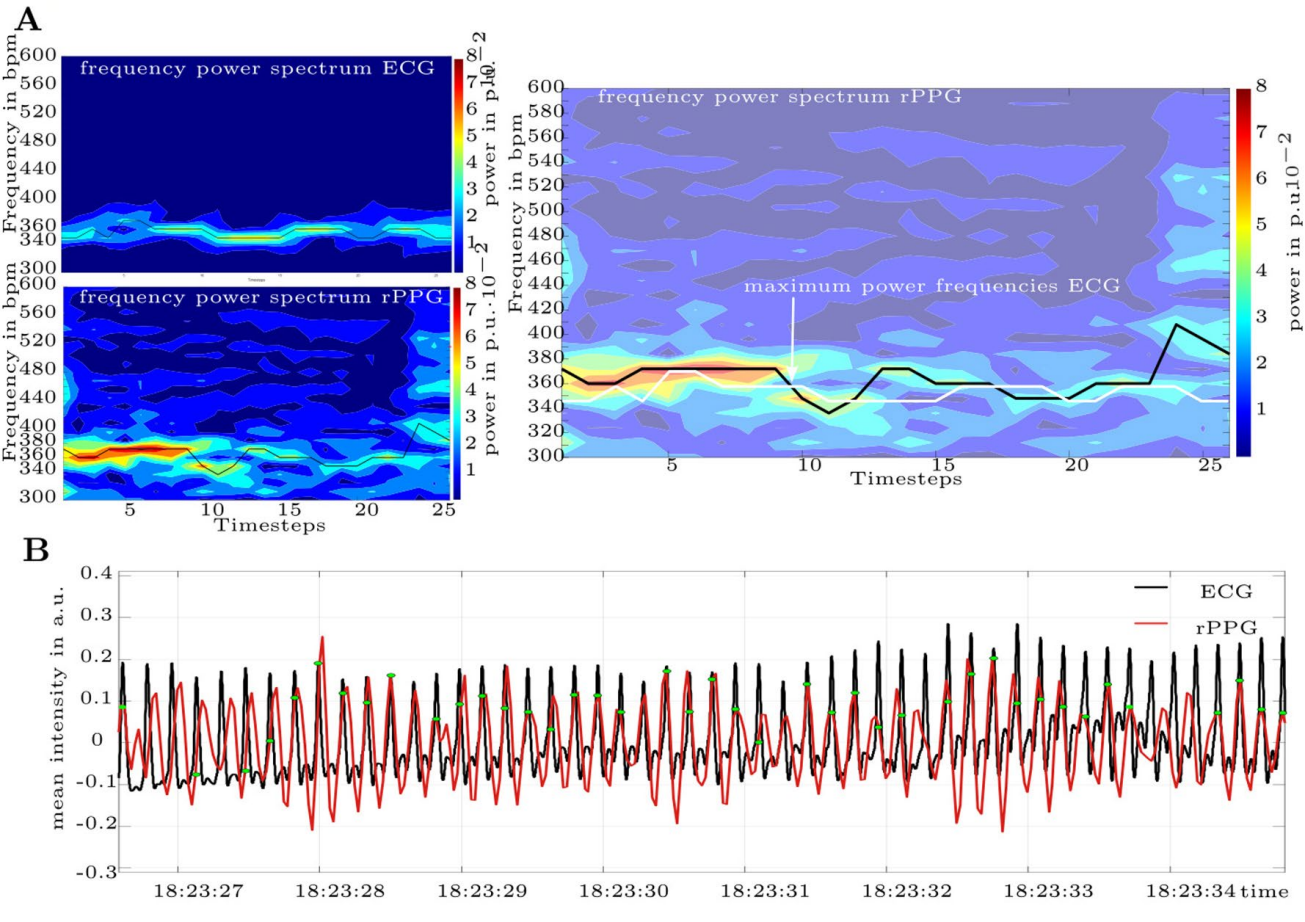
The frequency power spectrum of ECG and rPPG, and a segment of the respective signals in time domain. (**A**) The frequency power spectrum of ECG and rPPG. The plotted line indicates the frequencies with highest power within a timestep. HR of ECG: 355 bpm ± 1.5 bpm, HR of rPPG: 361 bpm ± 7 bpm (Mean and standard deviation). The rPPG signal is displayed in Fig. 6D. It should be noted that the last part of the spectrum, where the noise content is higher, originated from the last signal part, where the tracker failed. (**B**) A segment of the respective signals: ECG is scaled to rPPG, green dots indicate matching peaks within 0.05s tolerance.

In Fig. 7, the qualitative results from a side camera recording are shown (Fig. 6**D**)). The frequency power spectra of ECG and rPPG with a timestep of 5 s are depicted in Fig. 7A. The reference HR ranges from **337 bpm** to **377 bpm**, making the 30 s HR range **40 bpm**. The rPPG results in HR between **330 bpm** and **388 bpm**, hence a range of **58 bpm**. Although the rPPG spectrum was noticeably **noisier** than the ECG spectrum, with the highest power frequencies exhibiting a wider bandwidth, the detected HR still matched the reference, and the overall trend of the HR was discernible. The most error was introduced in the last 7 s, where the tracker failed (see Fig. 6D).

In Fig. 7B an approx. 8s-long segment of the denoised and filtered rPPG is shown, with the ECG-curve as reference. The matching peaks (with a tolerance of 0.05s) are indicated by dots. In this video 146 out of 203 peaks matched the reference ECG peaks.

**Fig. 8:**
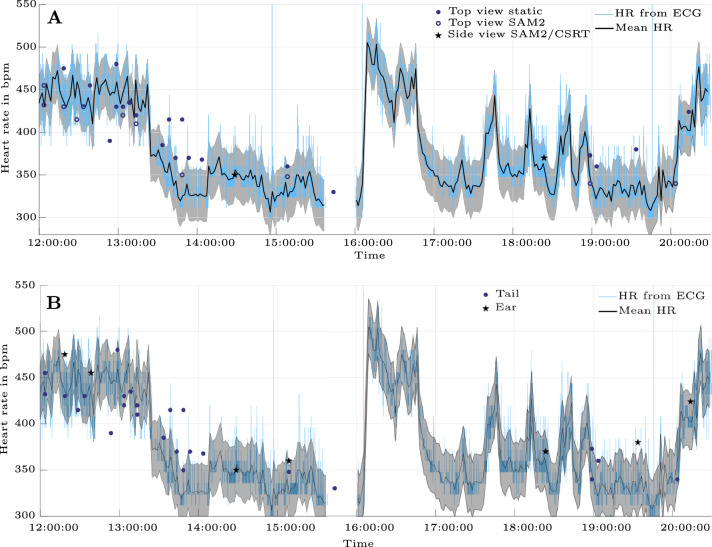
Time resolution in present dataset. Reference heart rate in 8.5 h timeframe and heart rates detected from rPPG. The shaded band marks the deviation of ± 30 bpm from the mean heart rate. (**A**) Top view, side view, static, SAM2, and CSRT. (**B**) Tail and ear.

Fig. 8 illustrates the reference HR over a period of 8.5 hours. The bold line represents the mean HR calculated for each 30 s signal segment. The black band indicates a range of ± 30 bpm around the mean HR. The achr extracted from rPPG is plotted for top view recordings and side view recordings (Fig. 8A), as well as for ear and tail (Fig. 8B).

The analysis was extended to two recordings each for animal 4 and animal 5, using the same preprocessing and parameter settings. HRs derived from rPPG were close to the reference: animal 4–recording 1: 357 bpm vs. 372 bpm ($\Delta$=−15 bpm), recording 2: 361 bpm vs. 372 bpm ($\Delta$=−11 bpm); animal 5–recording 1: 446 bpm vs. 446 bpm ($\Delta$=0 bpm), recording 2: 404 bpm vs. 408 bpm ($\Delta$=−4 bpm). Per animal, the mean absolute differences were 13 bpm (animal 4) and 2 bpm (animal 5). Across all four recordings, the mean absolute difference was 7.5 bpm.

## Discussion

In this study, different regions of interest, camera positions, and rPPG extraction methods were analyzed to investigate the feasibility of HR detection using classic signal processing techniques. This study represents the first investigation of contactless heart rate measurement in laboratory rats under realistic conditions. Our findings prove that the home cage concept is a suitable method, to extract the pulse signal and detect HR from video recordings. Within a ± 30 bpm tolerance the HR could be detected in up to **61.9%** of top view recordings, and in up to **81.8%** of side view recordings. However, certain limitations and factors affecting the accuracy were identified, which are discussed below.

Outliers can be attributed to factors such as frequency masking or insufficient pulse signal strength, rendering the pulse undetectable in those cases. Comparative analysis between side view and top view recordings revealed that the HR detection success rate was higher in side view recordings. The side view provides a better angle to capture subtle changes in skin color associated with blood flow, enhancing the quality of the signal extracted. In contrast, the top view may obscure these subtle changes due to a larger distance from the animal.

The pulse signal was detectable in both the red and blue channels. Using multiple color channels can improve the robustness of HR detection by compensating for signal loss or interference in a single channel.

Two tracking methods were compared: SAM 2 and the CSRT tracker. SAM 2 demonstrated advanced tracking capabilities, such as tracking objects even after they leave and re-enter the visible area. However, it was prone to errors in object detection, which could lead to inaccuracies in ROI selection and subsequent signal extraction. Due to the occasional lack of clear distinction of the body part within the RoI, the tracker’s effectiveness was constrained, thus unable to entirely mitigate motion artifacts. The CSRT tracker, while less advanced in continuous tracking, provided comparable results with lower computational cost. The advanced tracking capabilities of methods like SAM 2 come with increased computational cost. Real-time applications or scenarios with limited processing resources may benefit from using less computationally expensive methods like the CSRT tracker. Since a static RoI and SAM2 led to the same baseline wander in rPPG signals, motion artifacts could not be the sole explanation for noise. Lightning differences or camera inaccuracies could be another explanation.

The analysis revealed that, although no single body part consistently provided the strongest pulse signal, at least two usable RoIs were identified: the ear and the tail. Factors such as movement, lighting, and skin properties can influence signal strength. The small RoI size inherently leads to low signal strength and high susceptibility to noise. To optimize pulse signal detection, it is recommended that all suitable visible body parts be incorporated into the analysis. By leveraging multiple sources of data, this approach improves the overall reliability of HR detection.

Analyses of two further animals processed with the same parameters showed good accuracy to the reference, suggesting that the rPPG approach is not limited to one rat or one recording environment; larger cohorts will help quantify inter-animal variability.

This study faced several limitations affecting the practicality of HR extraction from video recordings. The signal extraction and HR identification within the frequency power spectrum were conducted manually. This manual process is time-consuming, not scalable, and introduces potential human error, limiting its application in real-time or large-scale settings. In this study, only video data from a single rat was analyzed to demonstrate the feasibility and proof of concept for video-based heart rate detection. This limitation may affect the generalizability of our findings to broader populations or different experimental conditions. Future studies should consider analyzing data from multiple subjects to validate and extend these preliminary results.

Contactless HR detection in laboratory animals has been explored using various technologies under ambient light conditions. In^18^, a radar-based measurement was utilized to detect cardiorespiratory movement in rats, achieving high accuracy with an average HR error of 0.33%. A Camera-based method was employed on anesthetized and constrained rats, reporting a mean Root Mean Square Error (RMSE) of 1.26 ± 0.87 beats per minute and a mean relative error of 0.27 ± 0.18%^23^. Additionally, contactless video-based measurements with comparable signal processing methods were used on anesthetized pigs, achieving a performance of 4.69 bpm in Mean Absolute Error (MAE), 6.43 bpm in RMSE^22^. In those studies the RoI was larger due to shaving or a larger animal, and recordings were made under daylight. Radio frequency near-field coherent sensing was applied to monitor vital signs of small conscious animals in their laboratory living quarters or natural habitats, successfully obtaining breathing and heartbeat curves but lacking reference data like ECG for validation^19^.

The method analyzing the sole of the foot reported in^24^ leveraged a 250 fps camera and bright, bedding-free cages, achieving achr identical to ECG but without continuous monitoring. The motion-based method in reported in^25^ tracked micro-motions to estimate respiration and then achr, reporting relative errors of 1.86%±0.82% (rats) and 1.20%±0.33% (mice) in well-lit settings.

In contrast, the present study focused on unrestrained rats under challenging lighting conditions with limited visible skin areas leading to small RoIs, and using consumer grade cameras. Therefore, less pixel containing the pulse signal were available, and more noise was introduced. Our Regions of Interest (ear, tail, paw) differ from sole-of-foot imaging and are chosen to remain usable despite bedding coverage. Despite these challenges, it achieved a MAE of **10.6 bpm** and a RMSE of **13.8 bpm**. While the accuracy is lower compared to studies with anesthetized or constrained animals, and well-lit environments the results are not directly comparable due to strongly differing conditions. Our accuracy (MAE = 10.6 bpm; RMSE = 13.8 bpm) translates to relative errors of roughly 1.8–3.5% and 2.3–4.6% across the 300–600 bpm range typical for rats. These values are comparable to MAEs reported in^25^ (7.8–10.5 bpm) under well-lit conditions, but do not reach the ECG-level agreement demonstrated in^24^.

This study demonstrates the feasibility of HR detection in freely moving animals under low light conditions. The results highlight the potential for contactless HR monitoring in more naturalistic and less controlled environments, and the time resolution achieved was sufficient for welfare assessment. Many physiological and behavioral paradigms in rodents involve heart-rate changes of tens of bpm (e.g., activity transitions, stress responses, circadian variation). Errors of 10–14 bpm are therefore sufficient to track trends and state changes under welfare-friendly home-cage conditions. However, the setup is not intended for beat-to-beat precision or heart rate variability/arrhythmia analysis; achieving those targets would likely require brighter visible illumination, higher frame rates, and/or specialized optics.

The present study focused on proving the concept of video-based heart rate detection in freely moving laboratory animals under realistic constraints, with accuracy that is consistent with prior video-based reports and with clear avenues for improvement. Future research will focus on automating the detection of furless skin areas and improving the tracking by employing deep learning approaches, which will improve the consistency of RoI selection and render manual intervention obsolete. Developing more robust denoising techniques, including the use of neural networks, is planned to consistently filter out noise and improve the quality of the extracted pulse signals. Efforts will also be directed towards automating HR detection within the frequency power spectrum to eliminate manual identification, thus making the process more scalable and efficient. Furthermore, improving the recording setup with better cameras and incorporating more side view recordings will provide higher-quality signals, enhancing HR detection success rates.

## Conclusion

This work presents an initial approach for video-based analysis of HR using remote rPPG in laboratory rats under low illumination conditions. Classic signal processing methods were employed to detect achr in a small dataset, demonstrating feasibility but acknowledging limitations in robustness. Our investigation suggests that classic signal processing methods can potentially extract achr from video recordings, indicating the viability of rPPG as a non-invasive alternative for achr monitoring. Challenges such as low signal strength and motion artifacts persist; however, careful selection of camera positions, RoIs, and tracking methods may help mitigate these issues. Further research is necessary to refine these techniques to enhance accuracy and reliability, which could eventually lead to practical applications in health monitoring and assessment of wellbeing.

## Data Availability

The datasets used and/or analysed during the current study available from the corresponding author on reasonable request. The dataset analysed during the current study are not publicly available yet due large dataset size of over 300 GB but are available from the corresponding author on reasonable request. We are working on a solution to make the data publicly available in the future.
